# Association between spinal cord compression ratio in magnetic resonance imaging, initial neurological status, and recovery after ventral slot in 57 dogs with cervical disc extrusion

**DOI:** 10.3389/fvets.2022.1029127

**Published:** 2023-01-06

**Authors:** Fernando Swiech Bach, Wilfried Mai, Luiz Felipe Silva Weber, José Ademar Villanova Junior, Leonardo Bianchi de Oliveira, Fabiano Montiani-Ferreira

**Affiliations:** ^1^Neurology Service, Clinivet Veterinary Hospital, Curitiba, Brazil; ^2^Comparative Ophthalmology Lab (LABOCO), Federal University of Paraná, Curitiba, Brazil; ^3^Department of Clinical Sciences and Advanced Medicine, Section of Radiology, School of Veterinary Medicine, University of Pennsylvania, Philadelphia, PA, United States; ^4^School of Veterinary Medicine, Small Animal Surgery Service, Pontifical Catholic University of Paraná (PUCPR), Curitiba, Brazil

**Keywords:** cervical herniation, intervertebral disc disease, disc extrusion, ventral slot, spinal surgery

## Abstract

This retrospective, unblinded, single rater study evaluated images obtained from magnetic resonance imaging (MRI) of dogs with cervical intervertebral disc extrusion before being submitted to ventral slot decompression (VSD). Dogs were re-evaluated systematically at 10 and 30 days after VSD. The objectives of this study were to investigate the associations between the following parameters: (1) The maximal spinal cord compression ratio (SCCR) as seen on transverse MRI and pre-surgical neurological status (NS) grade; we hypothesized that dogs with greater SCCR will have worse pre-surgical NS grade at presentation; (2) Pre-surgical NS grade and postoperative recovery; we hypothesized that worse pre-surgical NS grade will be associated with longer postoperative recovery time; (3) SCCR and postoperative recovery; we hypothesized that dogs with higher SCCR will have longer recovery time; (4) Location of extrusion (cranial *vs*. caudal) and initial NS grade and outcomes; we hypothesized that caudal cervical extrusion will have worse NS grade and longer time to recovery; (5) Longitudinal extension of ventral CSF signal loss on HASTE pulse sequence and NS grade and time to recovery; we hypothesized that dogs with longer HASTE CSF attenuation will have higher NS grade and longer time to recovery. There was no significant association between SCCR and NS grade, suggesting that this relationship in the cervical region is similar to what is observed in the thoracolumbar region, rejecting our first hypothesis. There was a significant difference between ambulatory tetraparesis dogs versus non-ambulatory tetraparesis dogs regarding complete recovery at 10 days: dogs with NS grade 1, 2, or 3 overall recovered faster than dogs with NS grade 4. However, there was no significant difference between these groups regarding complete recovery at 30 days, thereby accepting our second hypothesis at 10 days and rejecting it at 30 days. There was no correlation between SCCR and recovery time, rejecting our third hypothesis. Caudal cervical extrusion did not show higher NS grade or longer recovery time than cranial extrusion, rejecting our fourth hypothesis. CSF attenuation length ratio on HASTE images was not significantly correlated with NS grade but weakly correlate with post-surgical recovery time, partially accepting our fifth hypothesis.

## 1. Introduction

Intervertebral disc herniation (IDH) is the most common cause of spinal cord injury in dogs, with the cervical region being affected in approximately 15% of cases (1). According to the literature, the most commonly affected spaces in the cervical region are C2-C3 and C3-C4 (2). Small dogs, mainly chondrodystrophic, are predisposed to degeneration and calcification of the intervertebral discs, which are then prone to extrusion leading to varying degrees of spinal cord compression and injury (2). The amount of herniated disc material, the force of the extrusion and duration of compression are factors that may contribute to the severity of neurological deficits (1, 2). Any breed can be affected; however, the disease is more prevalent in Dachshunds, followed by Lhasa Apsos, Shih tzus, Beagles, French Bulldogs and Poodles (3). The classification of intervertebral disc disease has been recently reported in review articles, and generally, the term disc herniation can be used and interpreted as disc material exerting some degree of spinal compression (4). NS grade for cervical spinal cord injury ranges from cervical pain to tetraplegia, with neurogenic respiratory failure (Table 1) (5, 6). Cervical pain is the most common clinical sign in cervical disc herniation; tetraplegia is less frequent compared to paraplegia in thoracolumbar disc herniation. This is thought to be due to a larger vertebral canal to spinal cord ratio in the cervical region (7), although this anatomic regional variation appears to not be true in a recent study in French bulldogs (8), highlighting that other mechanisms may be contributing to severity of cord damage, which have not yet been elucidated.

**Table 1:** Neurological grading system for cervical spinal cord injury based on clinical signs.

**Neurological status grade**	**Clinical signs**
0	Normal
1	Hyperesthesia, walking with head down, shouting pain.
2	Ambulatory tetraparesis. Can walk with mild motor deficit or ataxia in the four limbs.
3	Ambulatory tetraparesis. Can walk with moderate motor deficit or ataxia in the four limbs.
4	No ambulatory tetraparesis (Tetraplegia). Cannot walk.
5	No ambulatory tetraparesis (Tetraplegia). Cannot walk with neurogenic hypoventilation.

The diagnostic imaging modality considered the “gold standard” for disc herniation is magnetic resonance imaging (MRI), as it can distinguish anatomical structures such as nerve roots, spinal cord parenchyma, epidural fat, cerebrospinal fluid and intervertebral disc layers (9). Ventral slot decompression (VSD) is the most widely used surgical treatment for ventral cervical disc herniation in dogs and has a good long-term prognosis (10). Risks related to this technique include hemorrhage, cardiac arrhythmias, cardiopulmonary arrest, aspiration pneumonia, spinal instability, and damage to adjacent structures such as the esophagus, trachea, vagosympathetic trunk and laryngeal recurrent nerve (10). The mortality rate for VSD in cervical herniation is 5.2% (11).

Although MRI provides direct visual assessment of the location, extent and degree of spinal compression, there are no well-defined guidelines for classifying the severity of spinal cord compression on cross-sectional images. Several studies have used some form of classification using spinal cord compression ratios (SCCR), especially in caudal cervical spondylomyelopathy (12, 13). Other authors have described cutoff points for the severity of compression such as mild compression, when < 25% of the vertebral canal's cross-sectional area is occupied by compressive material; moderate compression, from 25 to 50%; and severe compression, when >50% (14). There still are uncertainties regarding the associations between NS grade and SCCR, NS grade and postoperative recovery, SCCR and postoperative recovery. In a study including 33 dogs with cervical disc extrusion, Ryan et al. found a positive correlation between pre-surgical NS grade and degree of spinal cord compression, however, they found no correlation between degree of spinal cord compression and recovery (15). Da Costa et al. did not find correlation between degree of compression and NS grade in dogs with caudal cervical spondylomyelopathy (13), though the pathophysiology of this condition is different from acute disc extrusion.

In contrast to findings previously reported (15), we have the clinical impression that there is no strong association between degree of compression in dogs with cervical disc extrusion and neurologic status grade at presentation. Such association was not found for thoracolumbar disc extrusion (16), and these authors underscored the importance of other factors not reflected by the degree of physical compression, such as spinal cord contusion.

Several neurology veterinary texts, citing work by Waters et al. (17), list location of disc extrusion and pre-surgical neurological status as potential prognostic factors, stipulating that cranial cervical disc extrusion has a better outcome than caudal cervical extrusion and that dogs with non-ambulatory tetraparesis have worse outcomes than dogs with ambulatory tetraparesis. Here again, our clinical impression is different, and there appears to remain uncertainties as to whether these associations are true.

Finally, there is a paucity of studies evaluating the prognostic value of cerebral spinal fluid (CSF) attenuation length measured on HASTE pulse sequence in dogs with disc extrusion. Those studies evaluated this parameter in dogs with thoracolumbar disc extrusions (18, 19). To the authors' knowledge, there is no veterinary study looking at this in dogs with cervical disc extrusion.

The objectives of this retrospective, unblinded, single rater study were to evaluate images obtained from magnetic resonance imaging of dogs with cervical intervertebral disc extrusion before being submitted to ventral slot surgical decompression (VSD). Dogs were re-evaluated systematically at 10 and 30 days after surgery. The specific objectives were to investigate the associations between the following parameters: (1) The maximal spinal cord compression ratio (SCCR) as seen on transverse MRI images and pre-surgical neurological status (NS) grade; we hypothesized that dogs with greater SCCR will have worse pre-surgical NS grade at presentation; (2) Pre-surgical NS grade and postoperative recovery; we hypothesized that worse pre-surgical NS grade will be associated with longer postoperative recovery time; (3) SCCR and postoperative recovery; we hypothesized that dogs with higher SCCR will have longer recovery time; (4) Location of extrusion (cranial *vs*. caudal) and initial NS grade and outcomes; we hypothesized that caudal cervical extrusion will have worse NS grade and longer time to recovery; (5) Longitudinal extension of ventral CSF signal loss on HASTE pulse sequence and NS grade and time to recovery; we hypothesized that dogs with longer HASTE CSF attenuation will have higher NS grade and longer time to recovery.

## 2. Materials and methods

### 2.1. Inclusion criteria

The database of the neurology service at Clinivet Veterinary Hospital, Curitiba-PR, Brazil was searched over a period of 4 years (2017–2021) for dogs that presented with clinical signs of cervical spine dysfunction. Dogs were included if: (1) cervical disc extrusion was confirmed with MRI, (2) spinal cord decompression by ventral slot surgery was performed within a maximum of 2 weeks after onset of clinical signs, and (3) clinical follow-up was available at 10 and 30 days after surgery.

In dogs older than seven years of age, the preoperative work-up included thoracic radiographs and abdominal ultrasonography to rule out potential concomitant diseases that could contribute to poor patient recovery after spinal surgery. All included animals were deemed healthy enough to undergo VSD, with complete blood count and blood chemistry (including glucose, urea, creatinine, albumin, and alanine aminotransferase levels).

### 2.2. Clinical evaluation and data collection

The NS grade of each dog was scored 0–5 using a previously described (5, 6) grading system that is summarized in Table 1. A single observer (FB) determined the pre- and post- surgical NS grade. Full recovery was defined as being able to walk without ataxia and free of neck pain (NS grade 0). Partial recovery was defined as a post-surgical grade lower than the pre-surgical grade but still >0. Based on the NS grade at the 10- and 30-day recheck examinations, dogs were divided into 2 groups: short recovery (Group A, 1–10 days) and long recovery (Group B, 11–30 days). Additional data collected from the medical records included breed, sex, age. MR images were reviewed and the following data recorded: location of extrusion, size/degree of extrusion, and length of CSF signal loss on HASTE pulse sequence.

### 2.3. Imaging techniques and morphometric measurements

The diagnosis of cervical disc extrusion was confirmed by MRI (1.5 Tesla Avanto, Siemens, Erlangen, Germany), performed with patients anesthetized using intravenous propofol (B.Braun, Melsungen, Germany) at 6 mg/kg and placed in dorsal recumbency. The anesthesiologist stayed in the exam room to administer propofol as needed, based on respiratory frequency, heart rate, and palpebral reflex, until the end of MRI exam. Each exam took around 25 min to be completed. Pulse sequences included were sagittal plane T1-weighted (Repetition time (TR) / echo time (TE) 520/10 ms), T2-weighted (TR/TE 2,000/96 ms), HASTE (TR/TE 8,000/1,200 ms), STIR (TR/TE 3,890/69 ms), transverse plane T2-weighted (TR/TE 4,200/120 ms), dorsal plane STIR (TR/TE 3,000/31 ms) and sagittal /transverse plane (TR/TE 8.2/3.1 ms) post-contrast T1-weighted with fat suppression after administration of intravenous paramagnetic contrast medium (gadoteridol−0.5 mmol/ml, ProHance Bracco imaging, Germany) at a dose of 0.1 mmol/kg. All MRI sequences were performed with slice thickness that varied between 2 or 3 mm, the interslice gap was 0.2 mm, FOV area varied between 10 and 30 cm, depending of patient size. All MRI exams were performed in a human hospital.

**Figure 1:**
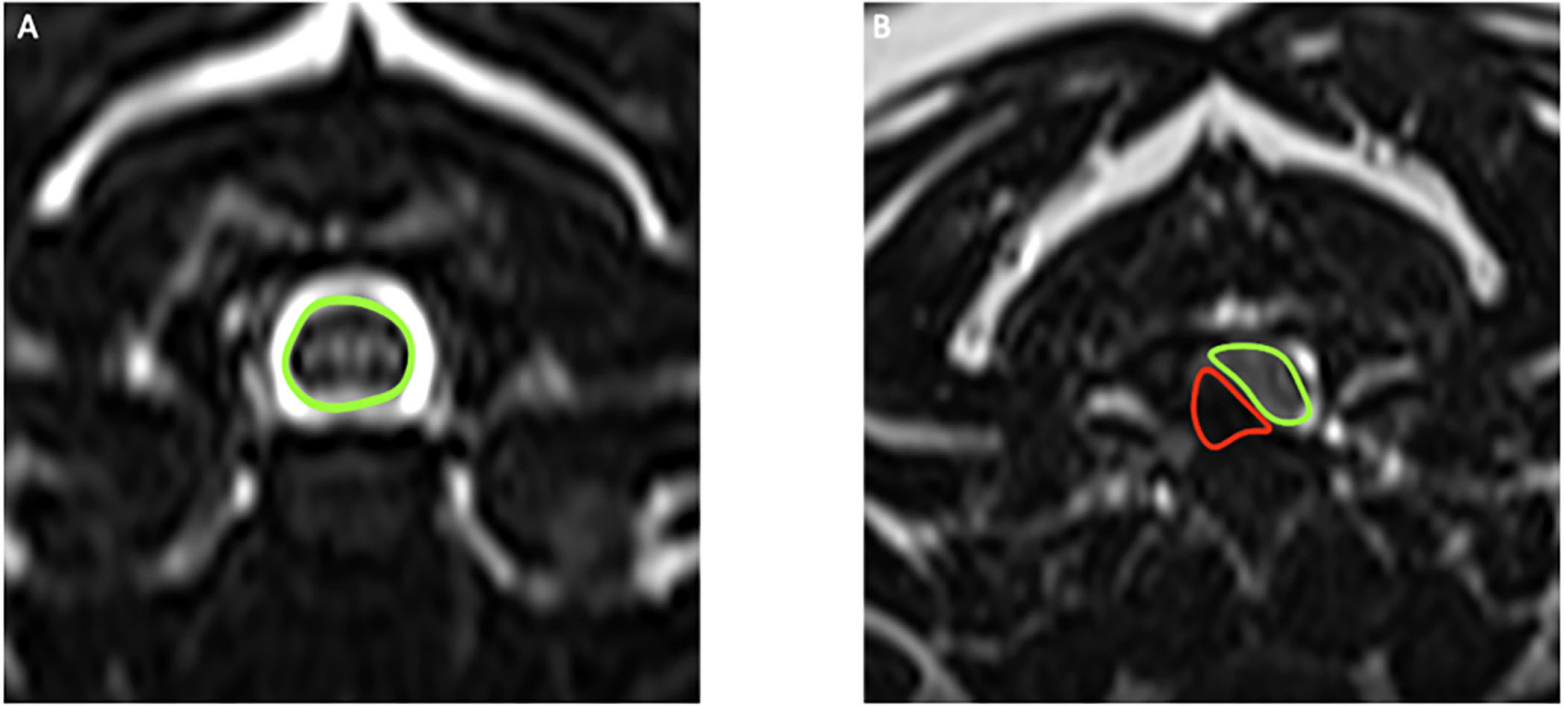
**(A)** T2-weighted transverse MRI of the cervical spine of a dog, with the uncompressed spinal cord highlighted in green. **(B)** T2-weighted transverse MRI of the compressed spinal cord (green) and extradural disc material (red) in a dog with intervertebral disc extrusion.

**Figure 2:**
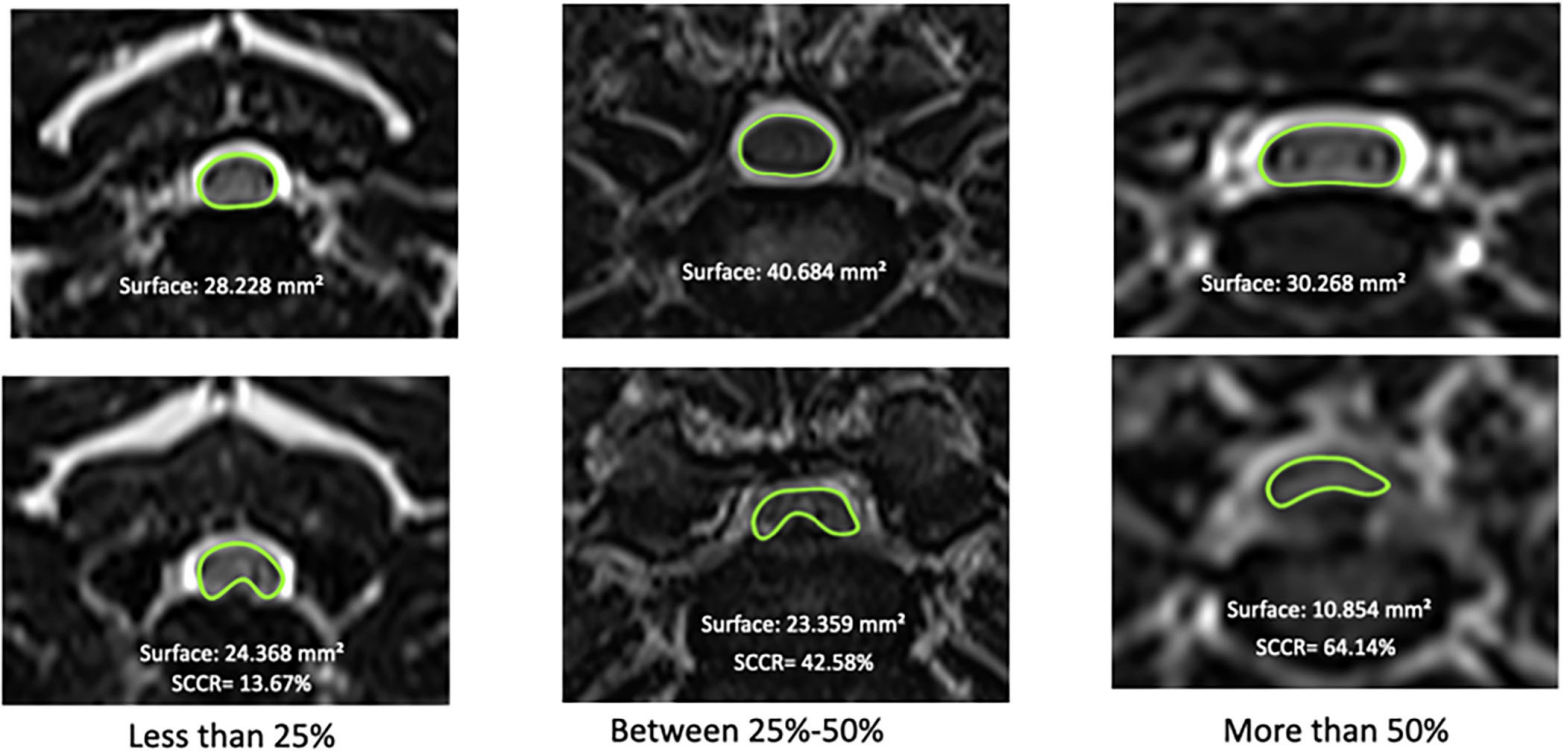
Examples of Spinal Cord Compression Ratio scores; mild, moderate and severe spinal compression based on measurement of surface area of the spinal cord at site of maximal compression *vs*. normal spinal cord at the disc space cranial to the compression. The top images represent the cranial normal disc space in the same dog with the spinal cord outlined in green; the images at the bottom are the sites of maximal spinal cord compression with the spinal cord outlined in green.

Measurements of SCCR was performed using transverse T2-weighted images at the level of the maximal spinal cord compression and were performed using the region of interest and surface area tool of a DICOM viewer (Horos for OS X, version 3–LGPL-3.0). To determine the SCCR, the surface area of the non-compressed spinal cord at the disc space cranial to the compression site was measured as well as the surface area of the cord at the site of subjective maximal compression, determined by visual assessment scrolling through the image series (Figure 1). The exact SCCR was then computed using the formula: SCCR = [(Surface area of non-compressed cord—Surface area of compressed cord)/Surface area of non-compressed cord] ^*^ 100. The exact SCCR was used to calculate correlations. Each case was also assigned to a categorical score of SCCR: mild compression for SCCR < =25%, moderate compression for SCCR >25% and < =50% and severe compression for SCCR >50% (12) (Figure 2).

**Figure 3:**
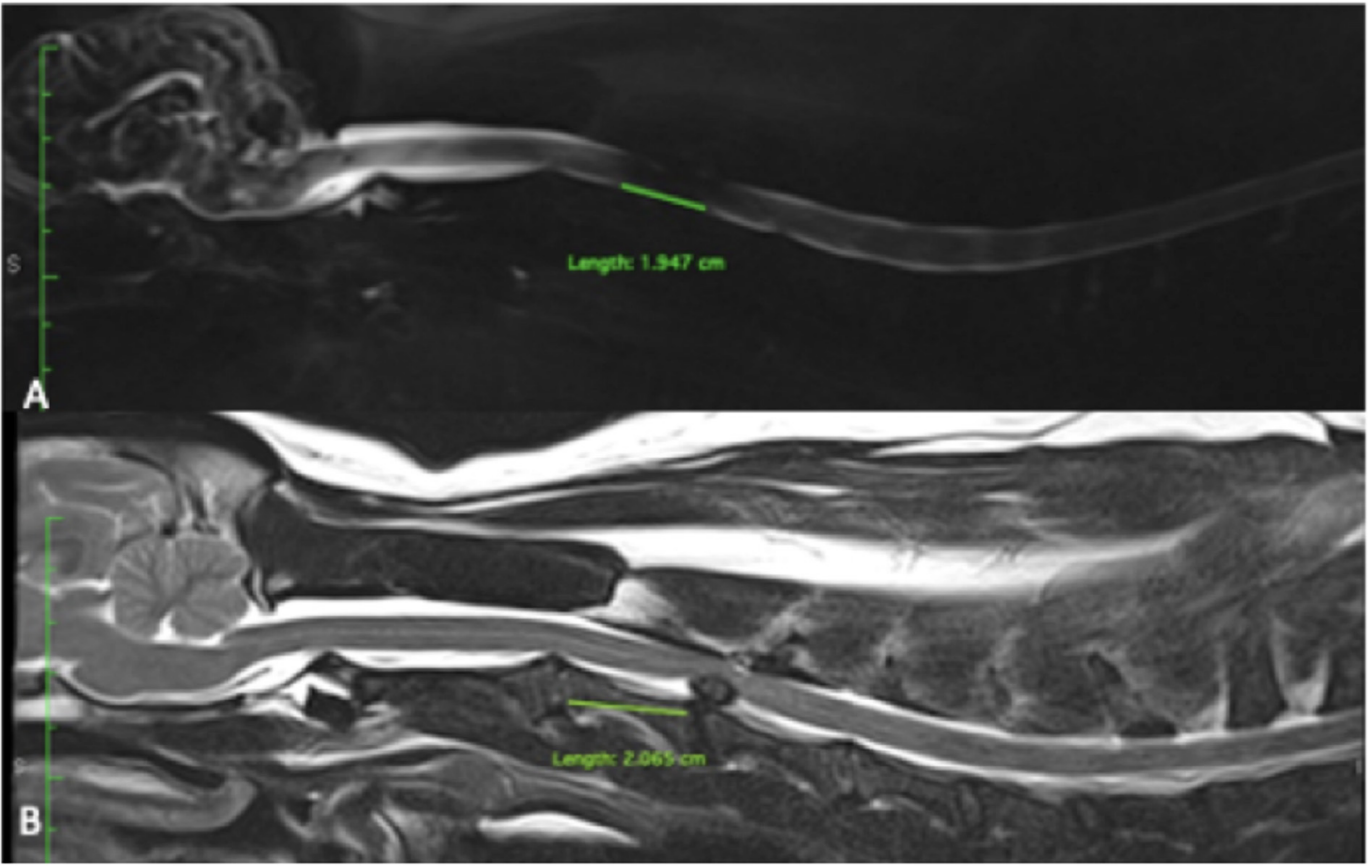
**(A)** Measure length of CSF attenuation between C3-C4 on HASTE. **(B)** Same dog measure of length of C3 and C3-C4 disk extrusion on T2 weight images. The ratio was measure by length of CSF attenuation on HASTE divided by length of C3 measure on T2 weight images.

The HASTE images were evaluated on sagittal images for presence or absence of CSF signal attenuation. When CSF signal attenuation was present, the maximal craniocaudal length of ventral CSF attenuation was measured in centimeters using the linear measurement tool of the same software as above. The length of the C3 vertebral body was measured in centimeters on sagittal T2-weighted images from the cranial extremity of vertebral body to the caudal extremity of vertebral body. A CSF length attenuation ratio was then calculated by dividing the length of CSF attenuation on HASTE by the length of C3 vertebral body (Figure 3).

To avoid interobserver discrepancies, all MRI images were prospectively evaluated by the same examiner (FB).

### 2.4. Anesthesia and surgical technique

Pre-anesthetic medication varied for each specific patient, but as a basic protocol, intravenous midazolam (0.25 mg/kg) and ketamine hydrochloride (1 mg/kg) were used followed by an intravenous bolus of propofol (5 mg/kg). Anesthesia was maintained using isoflurane as well as a continuous infusion of fentanyl (10 μg/kg/h). The same surgeon (FB), with substantial neurosurgical experience, performed all surgeries, using the technique described in the literature (20). The patient was positioned in dorsal recumbency, and the neck gently extended under a folded towel, which allows widening of the intervertebral disc spaces. The ventral cervical area was prepared for surgery by clipping the hair and local application of povidone-iodine. A sterile patch (Opsite Incise; Smith and Nephew, London, United Kingdom) was placed over the surgical field. Surgical material used for surgery was standard, including Surgairtome drill, Kerrison forceps, special curettes, burs and absorbable gelatin sponge (Surgifoam, Ethicon, USA). In a few dogs, additional disc fenestrations were performed at disc spaces where severe disc mineralization/dehydration or very minimal disc protrusion were noted on MRI, as a preventative measure to future clinical disc herniation.

### 2.5. Postoperative care and hospital discharge

The dogs were monitored for 48 h in the hospital's semi-intensive care unit. During this period, analgesia was performed by continuous infusion of fentanyl at a dose of 2 μg/kg/h. After 24 h, the infusion was discontinued and analgesia was performed with tramadol hydrochloride and sodium dipyrone subcutaneously at a dose of 3 and 30 mg/kg respectively, every 8 h, for 5 days. Prophylactic antibiotic therapy was performed by intravenous cephalothin administration at a dose of 30 mg/kg a few minutes before skin incision and every 6 h on the first postoperative day. Anti-inflammatory drugs were used if dogs showed signs of pain including vocalization, cervical stiffness or head ventroflexion after the surgery. In dogs that were receiving steroidal anti-inflammatory drugs prior to surgery, dexamethasone was administered at a dose 0.1 mg/kg subcutaneously once a day. In dogs that were receiving non-steroidal anti-inflammatory drugs prior to surgery, carprofen was administered at a dose at 2.2 mg/kg subcutaneously once a day. Steroidal and non-steroidal anti-inflammatory drugs were never used in combination.

All dogs remained in individual cages, with controlled room temperature, food and water. Each animal remained 2 days in the semi-intensive care unit and one day in the regular surgical wards until hospital release. At the time of discharge, owners were instructed to maintain activity restriction, monitor defecation and urination, in addition to light physical therapy exercises in tetraparetic dogs. The dogs were re-evaluated at 10 and 30 days by the same observer (FB) after hospital discharge.

### 2.6. Statistical analyses

Descriptive and inferential statistical analyses were performed. Spearman's rank-order correlation was used to analyze occurrence of associations between variables (SCCR *vs*. pre-surgical NS grade, SCCR *vs*. recovery time, CSF attenuation length ratio *vs*. NS grade and recovery time). Wilcoxon signed-rank test was used to compare overall and pairwise differences, respectively, between non-normally distributed paired data (pre-surgical NS grade, NS grade at 10 and 30 days after surgery). Recovery time analysis for both ambulatory (NS grades 1, 2, 3) and non-ambulatory groups (NS grade 4) was performed by Fisher's exact test. As a consequence of that a posteriori grouping, the “n” and statistical power were increased. Comparison between tetraparesis ambulatory and non-ambulatory dogs for complete recovery at 10 and at 30 days post-surgery was performed by Chi square test. Mann-Whitney test was used to compare NS grade and recovery times between dogs with cranial *vs*. caudal cervical disc extrusion. *P* < 0.05 were deemed significant.

## 3. Results

**Figure 4:**
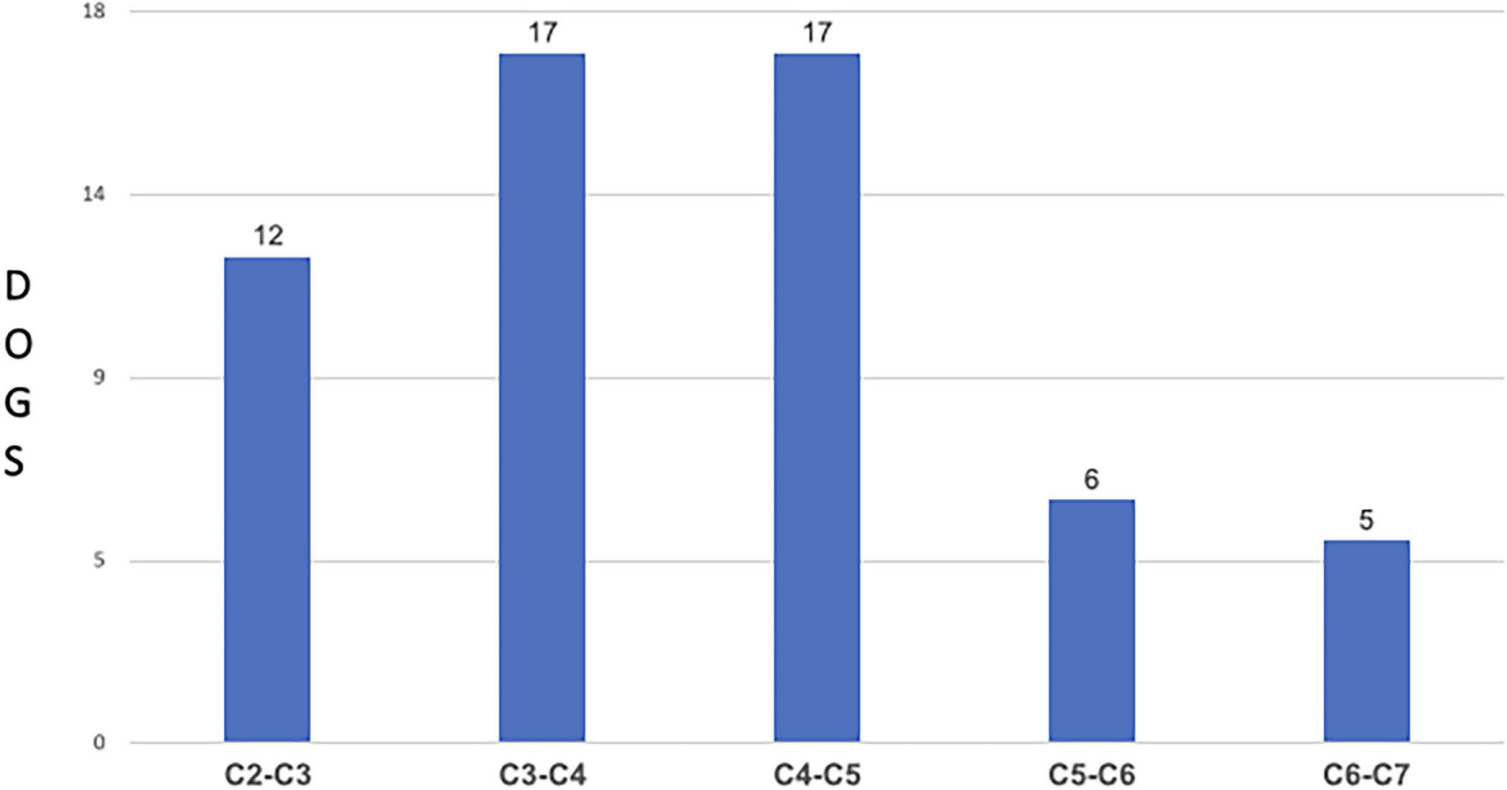
Distribution of Dogs and Spinal Cord Compression sites.

A total of 57 dogs fitted the inclusion criteria. The overall median age was 8.39 years (range 3 to 16 years). 31/57 dogs (54.39%) were males and 26/57 (45.61%) were females. There were 18 different breeds; the two most affected breeds were mixed breed 12/57 (21.05%), followed by French bulldog 10/57 (17.54%) (Table 2). The most affected disc space was C3-C4 in 17/57 (29.82%) and C4-C5 in 17/57 (29.82%) followed by C2-C3 in 12/57 (21.05%), C5-C6 in 6/57 (10.52%) and C6-C7 in 5/57 dogs (8.77%) (Figure 4). Slight lateralized disc extrusion was observed in 8/57 dogs (14.03%).

**Table 2:** Distribution of breeds with Cervical Intervertebral Disc Extrusion.

**Breed**	**Number**
Mixed-breed dog	12
French Bulldog	10
Dachshund	4
Yorkshire Terrier	4
Beagle	3
Lhasa Apso	3
Shit-tzu	3
Miniature Pincher	3
Rottweiler	3
Pekingese	2
Standard Poodle	2
Miniature Schnauzer	2
Spitz	1
Dobermann Pincher	1
Jack Russel	1
Labrador Retriever	1
Maltese	1
Pug	1
Total	57

After VSD, 44/57 dogs (77.19%) showed complete clinical recovery within 10 days (Group A) and 9/57 dogs (15.79%) showed complete recovery between 11 and 30 days (Group B). 3/57 dogs (5.26%) had an improved NS grade at 30 days but did not reach full recovery, and 1/57 dog had no improvement of NS grade at the 30-day recheck. Of the three dogs that had only partial recovery at 30 days, one dog was NS grade 4 pre-surgical and NS grade 2 at 30 days, one dog was NS grade 4 pre-surgical, and NS grade 3 at 30 days, and the last dog was NS grade 3 pre-surgical, and NS grade 1 at 30 days. Overall, 56/57 dogs (98.24%) had an improved NS grade within 30 days.

Surgery duration was consistently between 60 and 90 min. No significant complication (such as hemorrhage requiring transfusion, laryngeal paralysis, severe edema or hematoma in the muscles, subcutaneous tissues or skin around the neck) was observed. Surgical findings correlated with the imaging diagnosis in all cases. Additional disc fenestration was performed in 8/57 dogs (14.03%) at the following disc spaces: C3-C4 (four dogs), C4-C5 (two dogs) and C5-C6 (two dogs). All dogs in this study received a single VSD. No dogs had deterioration of their NS grade after surgery.

**Figure 5:**
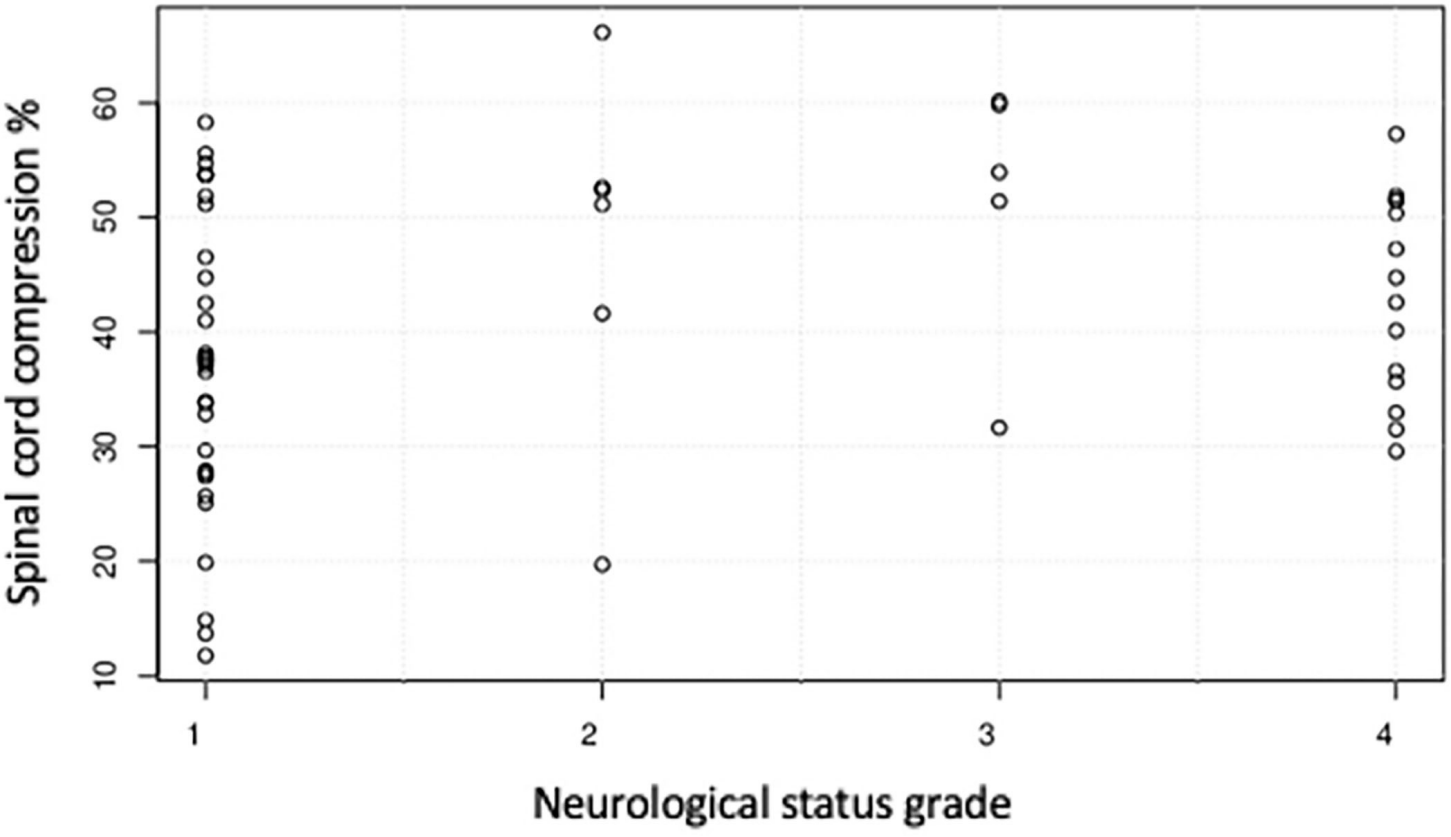
Relationship between spinal cord compression ratio and the neurological status grade of 57 dogs with cervical intervertebral disc disease before undergoing surgery.

**Figure 6:**
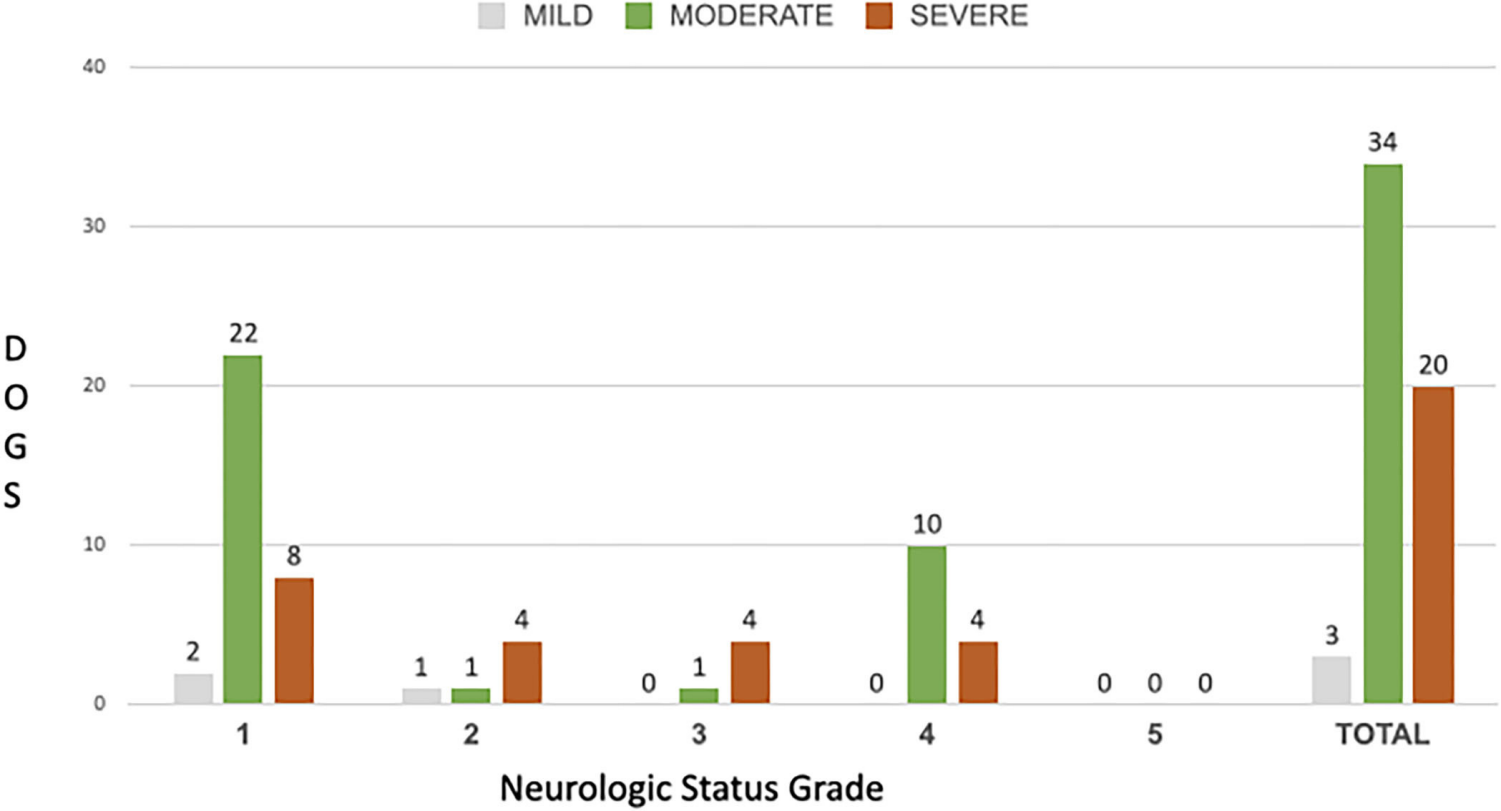
Distribution of Spinal Cord Compression ratio in each of the NS grades.

There was a weak (r_s_ 0.24) but non-significant (*p* = 0.07) positive correlation between pre-surgical NS grade and SCCR (Figure 5). At presentation, 32/57 dogs (56.14%) were NS grade 1, 6/57 (10.53%) were grade 2, 5/57 (8.77%) were grade 3, 14/57 (24.56%) were grade 4; all 57 dogs had intact deep pain perception in the thoracic limbs prior to surgery. The median spinal cord compression ratio was 41%, range 13–66%. Quantitative measurements of spinal cord compression severity by means of the SCCR calculation showed that 3/57 (5.26%) dogs had mild compression: 2/3 dogs (66.66%) were NS grade 1 and 1/3 (33.34%) was NS grade 2. 34/57 dogs (59.65%) had moderate compression, of which 22/34 (64.70%) were NS grade 1, 1/34 (2.94%) was NS grade 2, 1/34 (2.94%) was NS grade 3 and 10/34 (29.41%) were NS grade 4. 20/57 dogs (35.09%) had severe compression, of which 8/20 (40%) were NS grade 1, 4/20 (20%) were NS grade 2, 4/20 (20%) were NS grade 3 and 4/20 (20%) were NS grade 4 (Figure 6).

**Figure 7:**
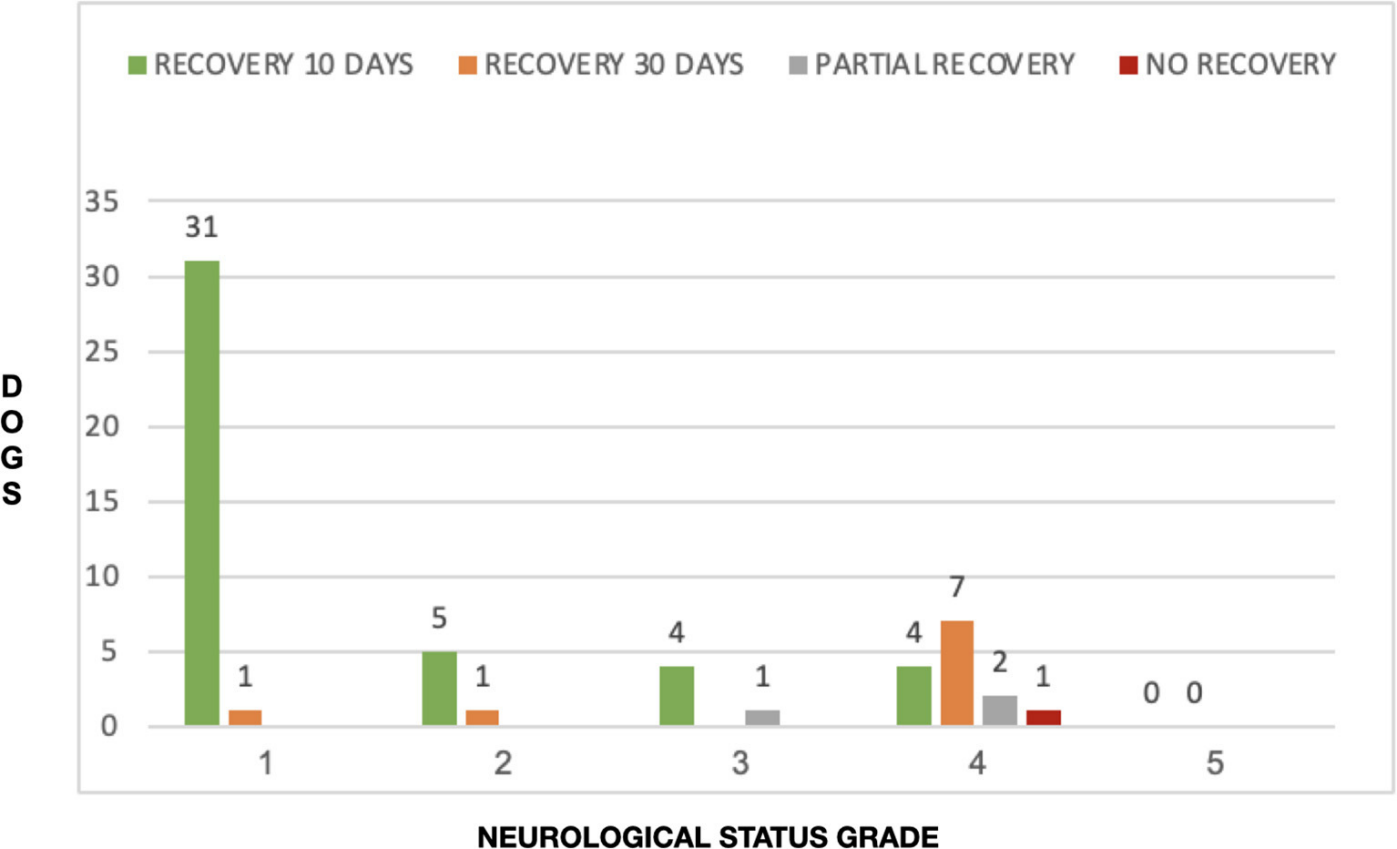
Distribution of recovery times in each of the NS grades.

When considering all ambulatory dogs (pre-surgical NS grade 1, 2, and 3), a total of 40/43 dogs (93.02%) recovered in 10 days and 2/43 (4.65%) recovered between 11 and 30 days; 1/43 (2.32%) did not completely recover. In this subgroup (ambulatory dogs), the proportion of dogs that had complete recovery at 10 days was significantly higher than those that recovered in 11–30 days (*P* < 0.001). Regarding non-ambulatory dogs (NS grade 4), 4/14 dogs (28.57%) had full recovery in 10 days and 7/14 dogs (50%) had full recovery in 11–30 days; 3/14 (21.43%) dogs did not make a complete recovery (two with partial improvement and one with no improvement). There was no significant difference regarding the proportion of non-ambulatory dogs (NS grade 4) that had a complete recovery within 10 days *vs*. the proportion of non-ambulatory dogs that had a complete recovery within 11–30 days (*P* = 0.5). However, when considering overall rate of complete recovery over the whole 30-day follow-up period, there was no significant difference (*P* = 0.40) between ambulatory dogs (42/43 complete recoveries, 97.7%) and non-ambulatory dogs (11/14 complete recoveries, 78.6%). There was an overall significant difference (*P* < 0.001) comparing pre-surgical NS grade with NS grade at 10 days and 30 days after surgery (Figure 7). In addition, there was a significant difference (*P* = 0.0031) between NS grade at 10 days after surgery *vs*. at 30 days after surgery.

**Figure 8:**
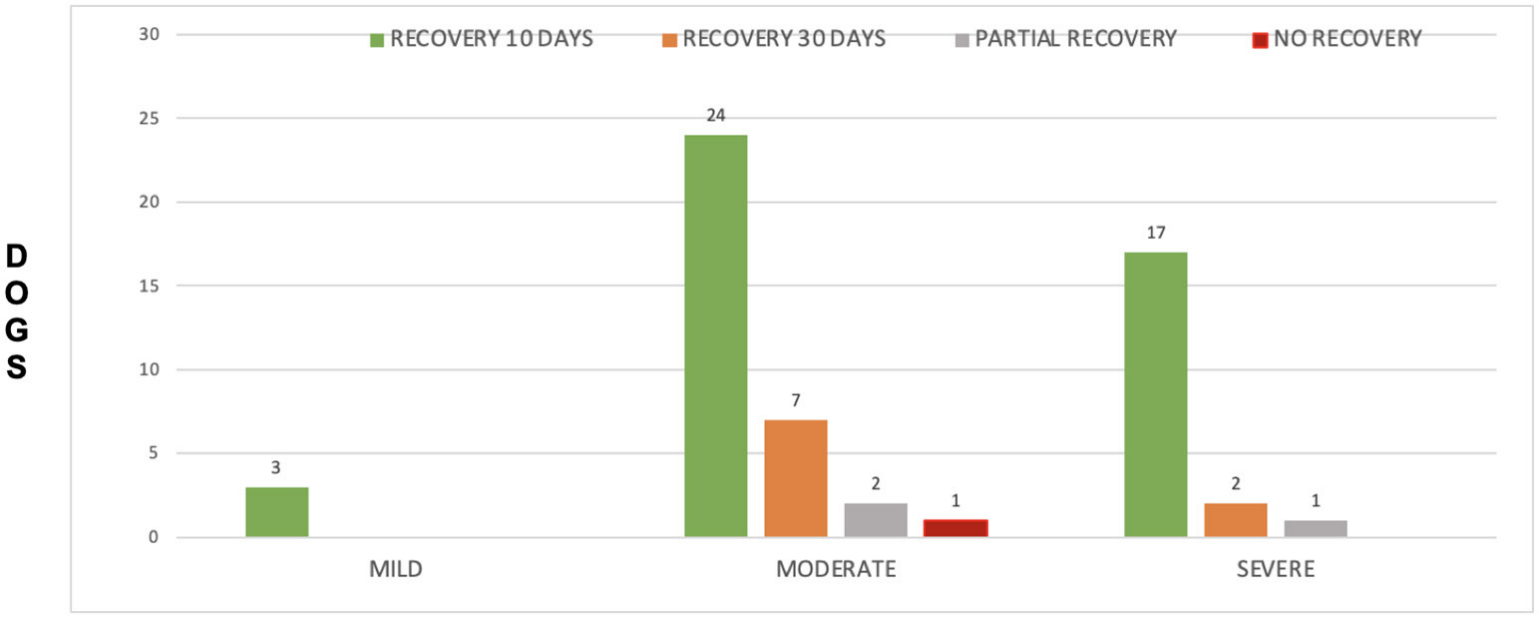
Distribution of recovery times in each of the Spinal Cord Compression ratios.

There was a non-significant correlation between categorical SCCR and post-surgical recovery time (Figure 8). SCCR was mild in three dogs, and all (100%) recovered completely in 10 days. Of the 34 dogs that had moderate SCCR, 24/34 (70.59%) had full recovery at 10 days, 7/34 (20.58%) had full recovery between 11 and 30 days and 2/34 (5.88%) had an improved NS grade at 30 days but did not achieve full recovery; 1/34 dog (2.95%) had no improvement in NS grade at 30 days. Finally, 20 dogs had severe SCCR; of those, 17/20 (85%) had complete recovery at 10 days after surgery, 2/20 (10%) had complete recovery between 11 and 30 days after surgery and 1/20 (5%) had improved NS grade at 30 days, but still had clinical signs (Figure 8). In all three groups of SCCR, the vast majority of dogs (44/57 or 77.2%) made a complete recovery in the 1-10 days post-surgery period.

**Figure 9:**
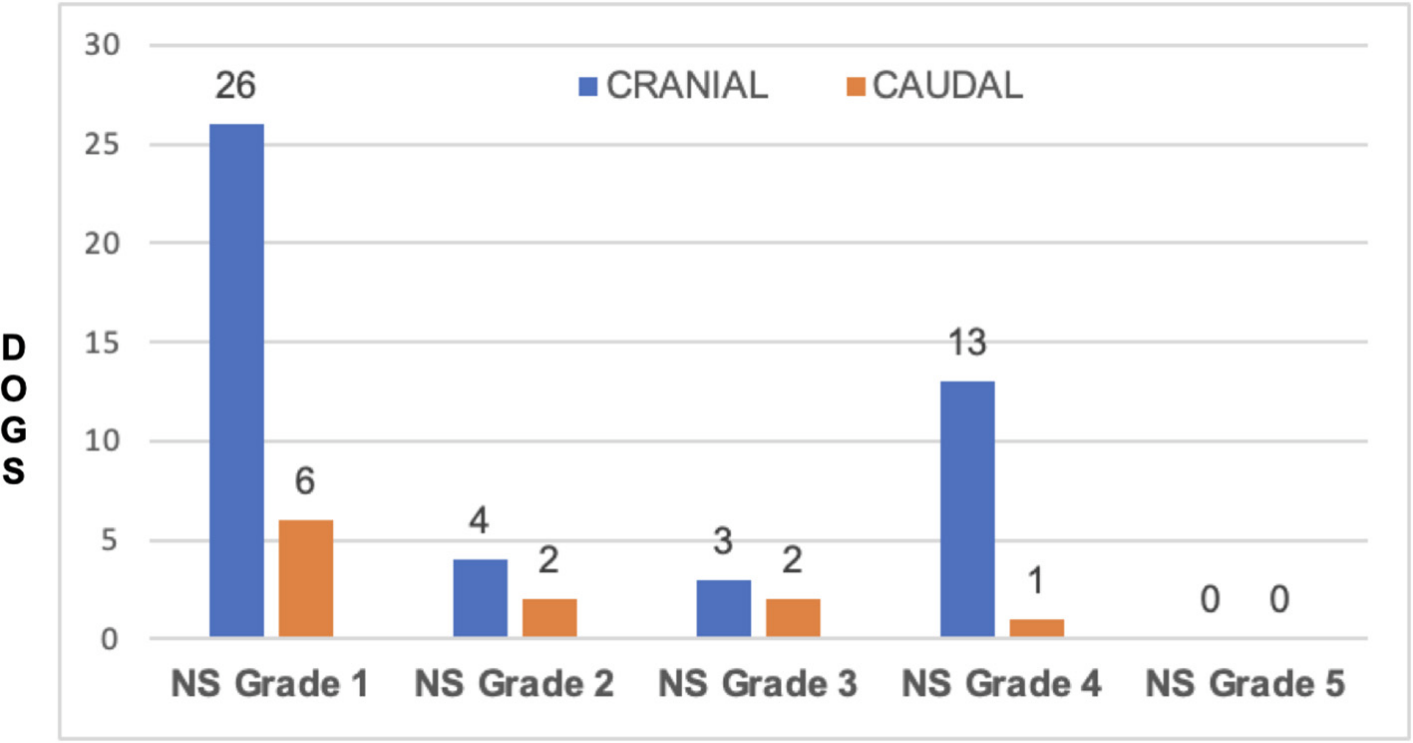
Distribution of Neurologic Status grade at cranial disc and caudal disc herniations.

**Figure 10:**
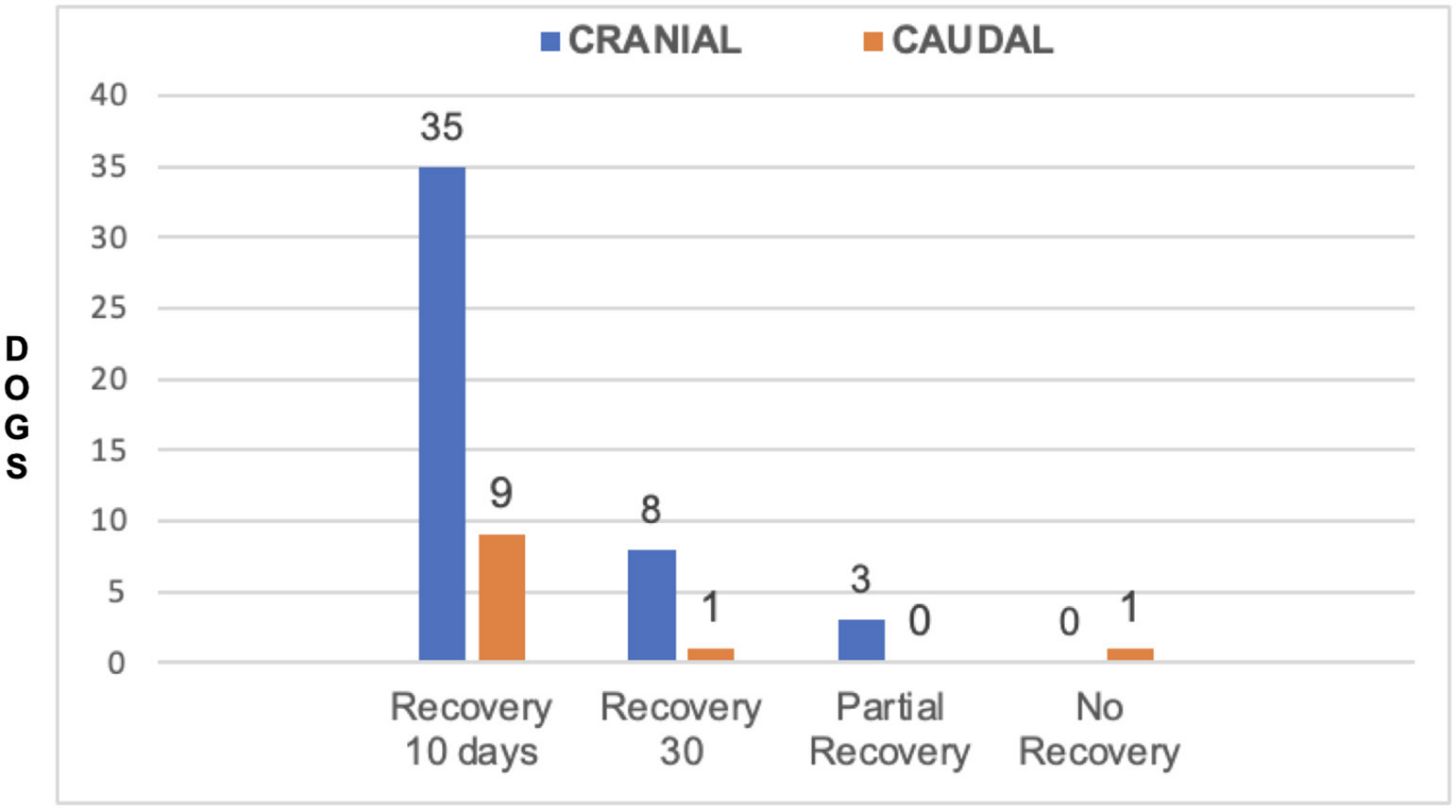
Distribution of recovery times at cranial disc and caudal disc herniations.

Forty-six dogs had VSD in cranial segments (C2-C3, C3-C4 and C4-C5). Of these, 33/46 (71.73%) dogs were NS grade 1, 2 or 3 and 13/46 (28.27%) dogs were NS grade 4. 35/46 dogs (76.08%) had full recovery in 10 days, 8/46 (17.40%) had full recovery in 11–30 days and 3 dogs did not recover completely. There were 11 dogs with VSD in the caudal cervical spine (C5-C6 and C6-C7). Of these, 10/11 dogs (90.90%) were NS grade 1, 2 or 3 and 1/11 (9.09%) was NS grade 4. Nine of these 11 dogs (81.8%) had full recovery in 1–10 days and 1 (9.09%) in 11–30 days; one dog did not have complete recovery at 30 days. There was no significant association between NS grade groups and site of extrusion (cranial *vs*. caudal) (*P* = 0.71) (Figure 9). In addition, there was no significant association between recovery time and site of extrusion (cranial *vs*. caudal) (*P* = 0.69) (Figure 10).

The median CSF attenuation ratio on HASTE images was 0.39 (range 0.1–0.9). There was a very weak (r_s_ 0.046) and non-statistically significant (*P* = 0.73) correlation with pre-surgical NS grade. There was a weak (r_s_ 0.26) positive correlation with recovery time, with a trend toward statistical significance (*P* = 0.05). There were three dogs (5.3%) that had only partial improvement in NS grade over the 30-day post-surgical period but did not make a full recovery. One dog had a C2-C3 disc extrusion with a moderate SCCR and a CSF attenuation ratio of 0.2. The pre-surgical NS grade was 3 and improved to NS grade 1 (minimal residual pain) within 30 days post-surgery. The other two dogs had disc extrusion at C3-C4: one had severe, and one had moderate SCCR; both had a CSF attenuation ratio of 0.3. Both dogs were NS grade 4 before surgery, and at 30 days after surgery, they had NS grades of 3 (dog with severe SCCR) and 2 (dog with moderate SCCR). Hence, both of these dogs regained ambulatory status. Finally, one dog (1.75%) had no post-surgical clinical improvement; this was a 12-year-old Labrador retriever with co-morbidities (diabetes mellitus); this dog had disc extrusion at C6-C7, moderate SCCR and a CSF attenuation ratio of 0.4, NS grade of 4 and lower motor neuron (LMN) signs in the thoracic limbs.

## 4. Discussion

The present study showed that dogs with severe SCCR due to cervical discs extrusion presented a great variation regarding clinical signs ranging from neck pain to non-ambulatory tetraparesis. This corroborates findings in the only similar study in the literature (15). Interestingly, our study showed a weak but non-significant correlation between SCCR and pre-surgical NS grade, which was different from the previously cited study (15). Nevertheless, our study showed that none of the patients with NS grade 3 and 4 had spinal compression classified as mild. There was a similar proportion of dogs presenting with NS grade 1 and NS grade 4 that had severe cord compression (25 and 28.57% respectively). Overall, the lack of correlation between compression severity and NS grade likely reflects the fact that other factors contribute to the severity of spinal cord injury, such as degree of spinal cord contusion. A similar amount of compression could be associated with a range of contusive spinal cord injuries, probably dependent upon the mechanical concussive forces exerted during the extrusion event. A comparison between the degree of cervical spinal compression and the relationship with clinical signs was investigated in Dobermans with caudal cervical spondylomyelopathy (CSM) (13). In that study the degree of spinal compression at MRI was more severe in normal dogs than in those with cervical hyperesthesia or mild ataxia (13). Doberman pinschers affected with CSM usually have chronic spinal cord injury secondary to disc protrusion and hypertrophy of the ligamentum flavum. However, the pathophysiology of CSM is different from spinal injury associated with acute disc extrusion, therefore these findings may not be applicable to dogs with acute disc extrusion. Penning et al. similarly observed no association between the NS grade and SCCR documented with MRI in dogs with thoracolumbar disc extrusion: that is expected, because spinal cord injury following intervertebral disc extrusion is a combination of concussive and compressive forces and further complicated by secondary mechanisms of injury (16). Overall, these studies and our findings suggest that there is no strong association between degree of compression and observed clinical signs in the cervical, and thoracolumbar regions. Ryan et al. (15) found in 33 dogs that severity of cervical spinal cord compression was significantly correlated with the presurgical NS grade (rs. 0.37 and *P* = 0.04), and reported a median SCCR on cross sectional images of 26% (range 11–71%). Our study revealed a weaker correlation between severity of compression and presurgical NS grade (r_s_ 0.24) and this did not reach statistical significance (*P* = 0.07). Possible explanations for this difference include a larger sample size and a bigger median SCCR in our study group (41%, range 13–66%).

Our study found that the majority of dogs with NS grade 4 recovered at 30 days while the majority of dogs with NS grade 1, 2, and 3 had a complete recovery at the 10 days recheck. These differences however must be interpreted with caution, since the group of dogs with NS grade 4 was small (14 dogs) compared to dogs with NS grades 1-2-3 (43 dogs). We used the recheck time points of 10- and 30-days post-surgery as these are the time intervals used as standard of care in our hospital (stiches removal at 10 days and last recheck at 30 days before ending cage restriction), and hence these time points were consistently available for data collection in all dogs of our study group. Indeed, some dogs across all NS grades improved completely as early as 1–5 days after surgery. Our study did not corroborate the findings in Waters' study, in which non-ambulatory tetraparesis (NS grade 4) was found to be a prognostic indicator for worse outcome (17). We found better outcomes in non-ambulatory dogs, with 78.6% making a complete recovery and 14.3% making a partial recovery. More recent and larger studies do not support Waters' findings either (2, 21) and are in agreement with our findings. Seim and Prata (22) reported good post-surgical recovery times in cervical disc herniations within 1 month. Our outcomes are overall slightly better than reported by Seim and Prata but this may reflect more accurate diagnostic methods (MRI *vs*. plain radiography or myelography) and the use of more modern surgical equipment.

In our study, there was no significant association between SCCR and recovery rates within 10 days (Group A) and within 11–30 days (Group B) post-surgery. Those findings are similar to the study by Ryan et al. (15) in 33 dogs with cervical disc extrusion, which found no association between the degree of spinal cord compression and the clinical outcome. Similarly, another study in the thoracolumbar spine by Penning et al. (16) did not find association between degree of spinal cord compression and surgical outcome. Again, this underscores the fact that other factors than the severity of the compression contribute to the outcome, such as the degree of spinal cord contusion, which is difficult to objectively quantify at MRI.

The most affected space was C3-C4 in 17/57 (29.82%) and C4-C5 in 17/57 (29.82%) followed by C2-C3 in 12/57 (21.08%). This is in agreement with other studies that showed that intervertebral disc extrusion is more common in the cranial cervical region (2, 15). Waters' study suggested that location of compression was a prognostic factor, and that cranial cervical lesions (C2-C3, C3-C4) were associated with greater likelihood of complete recovery than caudal cervical herniations (17). Our study did not find significant differences in post-surgical recovery between cranial and caudal disc extrusion Our findings corroborate results from a study by Hillman et al., which stated that site of disc extrusion was not a significant predictor for complete recovery; similar to our study, they found that dogs with cranial cervical disc extrusion did not have a better recovery compared to dogs that had caudal cervical disc herniations (21). In addition, our study did not find differences in NS grades between dogs with caudal *vs*. cranial cervical disc extrusion. This corroborates Cherrone et al.'s study that found no association between intervertebral disc space involved and ambulatory vs. non-ambulatory tetraparesis status (2). These findings were relayed in recent reviews of intervertebral disc disease in dogs (23). Overall, our findings seem to refute the notion that the site of cervical disc extrusion and severity of neurological signs are useful predictors of outcome.

One application of HASTE MRI pulse sequence is to obtain a “myelogram effect” on sagittal images, using heavy T2-weighting that highlights the signal from the CSF. This sequence is excellent for a rapid evaluation of the subarachnoid space, and in dogs with acute disc herniation, allows a quick localization of areas of extradural spinal cord compression and/or cord swelling from contusion and edema (14). HASTE pulse sequence was reported to be a potential prognostic tool in dogs with thoracolumbar disc extrusion and found that dogs with longitudinal extension of loss of CSF signal of < 7.4 times the length of L2 were less likely to develop progressive myelomalacia (18). In our study, in dogs with cervical disc herniation, we did not observe a significant correlation between extent of loss of CSF signal on HASTE images and NS grade at presentation which corroborates findings by Khan et al. in dogs with thoracolumbar disc herniation (19). Our study found a weak positive correlation with recovery time. Nevertheless, this information must be interpreted with caution, since only four dogs did not have a full recovery (three partial recoveries and one that did not recover). Therefore, a direct comparison to the clinical features observed in the group of dogs analyzed here is not possible.

In this study, chondrodystrophic breeds such as the French bulldog, Dachshund, Lhasa Apso, Shih tzu, Pekingese, Beagle and Poodle had a high prevalence, together making up 47% of the study group. This corroborates findings in the study by Ryan et al., which had 42% of chondrodystrophic breeds in their study group (15). The average age in our group was 8.4 years, in agreement with previous studies where the mean age was 8.6 years (15).

All 57 dogs had VSD. We performed disc fenestration in 8/57 dogs (14.03%) at the same time as VSD surgery. Fenestration was performed in dehydrated discs (as seen on MRI by complete loss of T2-weigthed signal) or in areas of minimally compressive disc protrusion but it was beyond the scope of this study to evaluate recurrence rate with regards to the performance of fenestration surgery. The choice of disc space to perform the ventral slot was based on the site of the most severe spinal compression observed at MRI. No dogs had more than one VSD in this study. No dog was re-imaged or needed re-intervention in the 30-day post-operative period. The only dog that was not improved was a 12-year-old Labrador with diabetes mellitus that had an SCCR of 41.13%. Ideally, re-imaging this dog would have been indicated but for financial reasons the owner decided to not repeat MRI. This particular dog had a C6-C7 disc extrusion and presented with lower motor neuron signs in the thoracic limbs and a NS grade of 4. Lower motor neuron lesions usually carry a worse prognosis than upper motor neuron lesions (17). However, only 10 other dogs had caudal disc extrusion (C5-C6 or C6-C7) and because they had NS grade 1 (*n* = 6), 2 (*n* = 2) or 3 (*n* = 2) with only discrete or moderate motor deficits, an accurate determination of lower *vs*. upper motor neuron lesions was not possible. Therefore, it was not feasible in our study to assess the influence of upper *vs*. lower motor neuron signs on recovery.

Ryan et al. (15) observed deterioration of the NS grade in 3/33 (9.09%) dogs with cervical disc herniation after decompressive surgery. In another study of 173 dogs with thoracolumbar disc herniations, Forterre et al. observed clinical deterioration within 1–10 days after hemilaminectomy in 5.8% of cases (24). We did not observe clinical deterioration in our study. Of the three dogs that had an NS grade improvement but did not show full recovery, two had moderate and one had severe SCCR. Post-surgically, one dog had minimal residual neck pain and two dogs were walking with mild ataxia and were pain-free. Due to financial reasons, no re-imaging procedures were performed to evaluate any remaining disc material. In these dogs, the median CSF length attenuation ratio measured on HASTE images was 0.2–0.3, i.e. lower than the median across all 57 dogs (0.39 times the length of C3).

In the literature, mortality associated with VSD surgery in dogs is 5.2% (10). We observed no mortality in our study group.

The inclusion criteria were VSD within a maximum period of 2 weeks from onset of clinical signs. A systematic review of the literature was recently reported by the Canine Spinal Cord Injury Consortium and found no consensus that duration of signs influences outcome, though found some evidence that it may influence the speed of recovery (25). Two studies that evaluated outcomes in cervical disc extrusion, Ryan et al. (15) and Hillman et al. (21) had a wide range of duration of clinical (1 day to 1 year). We decided to use a cut point of 2 weeks to make our study group more homogeneous.

This study has some limitations. The same surgeon reevaluated the dogs at 10 and 30 days, which could potentially lead to some judgment bias; a separate clinical assessment by another clinician blinded to the imaging and surgical results would have been optimal, however this was not possible given the retrospective nature of this study. Our observation time points for post-surgical recovery were set at 10 and 30 days but recovery may have occurred earlier than these delays in many dogs. These time points were chosen because they are consistent recheck points for all dogs undergoing VSD surgery at our hospital (removal of stitches at 10 days after surgery and last clinical recheck before lifting exercise restriction at 30 days after surgery). Although the time between clinical onset and VSD was not consistent across all dogs, all had surgery within 2 weeks after clinical onset so the variability is considered limited. We had a limited number of non-ambulatory dogs (14 dogs); a future study with higher number of non-ambulatory dogs should be appropriate. No neuroimaging after surgery was performed, to see how much disc material remained; although re-imaging is not a very common procedure in veterinary ventral slot surgery, it certainly should be considered in dogs that do not improve after surgery.

## 5. Conclusions

Ventral Slot Decompression is a safe technique for the treatment of ventral cervical disc extrusions and, when properly performed, has an excellent prognosis. The lack of significant association between SCCR and NS grade suggests that this relationship in the cervical region is similar to that observed in the thoracolumbar region, rejecting our first hypothesis. Regarding pre-surgical NS grade and recovery time after VSD, a significantly larger proportion of ambulatory dogs (NS grades 1-2-3) have a short recovery time (< 10 days) compared to non-ambulatory tetraparetic dogs (NS grade 4). However, at 30 days after surgery, there was no significant difference between ambulatory tetraparesis vs. non-ambulatory tetraparesis dogs regarding complete recovery, partially accepting our second hypothesis. The degree of spinal cord compression as measured by MRI-derived SCCR was not associated with the recovery time after surgery, rejecting our third hypothesis. Caudal cervical extrusion did not show higher NS grades or a longer recovery time than cranial extrusion, rejecting our fourth hypothesis. CSF attenuation length ratio on HASTE images was not significantly correlated with NS grade, but weakly correlated with post-surgical recovery time, only partially accepting our fifth hypothesis.

## Data availability statement

The original contributions presented in the study are included in the article/supplementary material, further inquiries can be directed to the corresponding author.

## Ethics statement

The animal study was reviewed and approved by Comitê de Ética de Uso de Animais da Pontifícia Universidade Católica do Paraná (PUCPR), Brazil. Written informed consent was obtained from the owners for the participation of their animals in this study.

## Author contributions

FB performed the neurologic examinations, MRI and images analyses, surgeries, and reevaluated all patients. WM improved English syntax and grammar, provided input as far as the methods, and analysis of results and discussion. LW helped with manuscript writing and reference organization. JV helped with manuscript writing. LB helped with clinical data acquisition. FM-F principal investigator, helped with manuscript writing, and statistical analyses. All authors contributed to the article and approved the submitted version.

## References

[1] Gage ED. Incidence of clinical disc disease in the dog. J Am Anim Hosp Assoc. (1975) 11:135–8.

[2] Cherrone KL Dewey CW Coastes JR Bergman RL A. retrospective comparison of cervical intervertebral disk disease in nonchondrodystrophic large dogs versus small dogs. J Am Anim Hosp Assoc. (2004) 40:316–20. 10.5326/0400316 15238562

[3] Brisson BA Moffatt SL Swayne SL Parent JM. Recurrence of thoracolumbar intervertebral disk extrusion in chondrodystrophic dogs after surgical decompression with or without prophylactic fenestration: 265 cases (1995–1999). J Am Vet Med Assoc. (2004) 224:1808–14 10.2460/javma.2004.224.1808 15198267

[4] Fenn J Olby NJ. Classification of intervertebral disc disease. Front Vet Sci. (2020) 7:579025. 10.3389/fvets.2020.579025 33134360 PMC7572860

[5] Rossmeisl JH Lanz OI Inzana KD Bergman RL A. modified lateral approach to the canine cervical spine: procedural description and clinical application in 16 dogs with lateralized compressive myelopathy or radiculopathy. Vet Surg. (2005) 34:436–44. 10.1111/j.1532-950X.2005.00066.x 16266334

[6] Rusbridge C Wheeler SJ Torrington AM Pead MJ Carmichael S. Comparison of two surgical techniques for the management of cervical spondylomyelopathy in Dobermans. J Small Animal Pract. (1998) 39:425–31. 10.1111/j.1748-5827.1998.tb03749.x 9791829

[7] Coates JR. Intervertebral disk disease. Vet Clin North Am Small Anim. (2000) 30:77–110. 10.1016/S0195-5616(00)50004-7 10680210

[8] Silva S Aude Genain M Khan S. Gauton JR, Freeman P. The spinal cord-to-vertebral canal area ratio measured with computed tomography is lower in the thoracolumbar than the cervical region in French bulldogs. J Am Vet Med Assoc. (2022) 1–4. 10.2460/javma.22.06.0266 36006917

[9] da Costa RC de Decker S Lewis MJ Volk H. Diagnostic imaging in Intervertebral Disc Disease. Front Vet Sci. (2020) 7:588338. 10.3389/fvets.2020.588338 33195623 PMC7642913

[10] Rossmeisl JH White C Pancotto TE Bays A Henao-Guerrero PN. Acute adverse events associated with ventral slot decompression in 546 dogs with cervical intervertebral disc disease. Vet Surg. (2013) 42:795–806. 10.1111/j.1532-950X.2013.12039.x 23980621

[11] Posner LP Mariani CL Swanson C Asawaka M Campbell N King A. Perianesthetic morbidity and mortality in dogs undergoing cervical and thoracolumbar spinal surgery. Vet Anaesth Analg. (2014) 41:137–44. 10.1111/vaa.12127 24588930

[12] Provencher M Habing A Moore SA Cook L Phillips G da Costa RC. Kinematic magnetic resonance imaging for evaluation of disc-associated cervical spondylomyelopathy in doberman pinschers. J Vet Internal Med. (2016) 30:1121–8. 10.1111/jvim.13981 27239003 PMC5089627

[13] da Costa RC Parent JM Partlow G Dobson H Holmberg DL LaMarre J. Morphologic and morphometric magnetic resonance imaging features of Doberman Pinschers with and without clinical signs of cervical spondylomyelopathy. Am J Vet Res. (2006) 67:1601–12. 10.2460/ajvr.67.9.1601 16948609

[14] Mai W. “Normal MRI spinal anatomy, degenerative disc disease, and disc herniations,” in *Diagnostic MRI in Dogs and Cats*, Mai, W. (ed). Boca Raton, FL: CRC Press. p. 411–446.

[15] Ryan TM Platt SR Llabres-Diaz FJ McConnell JF Adams VJ. Detection of spinal cord compression in dogs with cervical intervertebral disc disease by magnetic resonance imaging. Vet Record. (2008) 163:11–5. 10.1136/vr.163.1.11 18603629

[16] Penning V Platt SR Dennis R Cappello R Adams V. Association of spinal cord compression seen on magnetic resonance imaging with clinical outcome in 67 dogs with thoracolumbar intervertebral disc extrusion. J Small Animal Pract. (2006) 47:644–50. 10.1111/j.1748-5827.2006.00252.x 17076787

[17] Waters DJ. Nonambulatory tetraparesis secondary to cervical disk disease in the dog. J Am Anim Hosp Assoc. (1989) 25:647–53. 10.5326/0450155 19570897

[18] Gilmour LJ Jeffery ND Miles K Riedesel E. Single-shot turbo spin echo pulse sequence findings in dogs with and without progressive myelomalacia. Vet Radiol Ultrasound. (2017) 58:197–205. 10.1111/vru.12463 27977066

[19] Khan S Freeman P. HASTE MRI sequence findings correlate with loss of deep pain perception in dogs with thoracolumbar disc extrusion. Vet Med Sci. (2022) 1–5. 10.1002/vms3.974 36303241 PMC10029906

[20] Sharp N Wheeler S. Small Animal Spinal Disorders: Diagnosis and Surgery. 2nd ed. Maryland Heights, MO: Mosby Elsevier. (2005). 10.1016/B978-0-7234-3209-8.50008-X

[21] Hillman RB Kengeri SS Waters DJ. Reevaluation of predictive factors for complete recovery in dogs with nonambulatory tetraparesis secondary to cervical disk herniation. J Am Anim Hosp Assoc. (2009) 45:155–63. 10.5326/0450155 19570897

[22] Seim H Prata R. Ventral decompression for the treatment of cervical disk disease in the dog: a review of 54 cases. J Am Anim Hosp Assoc. (1982) 18:233–40.

[23] Brisson BA. Intervertebral disc disease in dogs. Vet Clin North Am Small Anim. (2010) 40:829–58. 10.1016/j.cvsm.2010.06.001 20732594

[24] Forterre F Gorgas D Dickomeit M Jaggy A Lang J Spreng D. Incidence of spinal compressive lesions in chondrodystrophic dogs with abnormal recovery after hemilaminectomy for treatment of thoracolumbar disc disease: A prospective magnetic resonance imaging study. Vet Surg. (2010) 39:165–72. 10.1111/j.1532-950X.2009.00633.x 20210963

[25] Olby NJ da Costa RC Levine JM Stein VM The The canine spinal cord injury consortium (CANSORT SCI). Prognostic factors in canine acute intervertebral disc disease. Front Vet Sci. 7:596059. 10.3389/fvets.2020.596059 33324703 PMC7725764

